# How did the ‘whistle-to-whistle’ ban affect gambling advertising on TV? A live football matching study

**DOI:** 10.1080/16066359.2024.2355183

**Published:** 2024-05-20

**Authors:** Ellen McGrane, Robert Pryce, Luke Wilson, Matt Field, Elizabeth Goyder

**Affiliations:** a Sheffield Centre for Health and Related Research (SCHARR), University of Sheffield, Sheffield, UK; b Department of Psychology, University of Sheffield, ICOSS Building, Sheffield, UK

**Keywords:** Gambling, advertising, sport, football, policy, economics

## Abstract

**Background:**

In 2019, the gambling industry introduced a voluntary partial advertising ban during live sports broadcasts in the United Kingdom known as the ‘whistle-to-whistle’ ban. This study explores the change in television advertising around live football games following the introduction of this ban.

**Methods:**

Inverse Probability Weighted (IPW) matching models identified the change in the frequency of advertising associated with the implementation of restrictions in each section, and across the entire duration, of a live football game. Data on UK television schedules (Concise Media, TVSportsGuide) and gambling advertising (Nielsen Media) covered 3 months (1^st^ September to 1^st^ December) pre (2018), and post-ban (2019). There were 1049 live football games across the period studied: 468 in 2018 and 581 in 2019.

**Results:**

The implementation of the ban was associated with a reduction in advertising (2.3 advertisements per-programme (*p* < 0.001, CI [−2.75, −1.84])), driven predominantly by reductions during half-time (2.18 advertisements per-programme (*p* < 0.001, CI [−2.32, −2.04])). It was associated with an increase in advertisements (0.34 advertisements per-programme (*p* < 0.001, CI [0.09,0.59])) during the pre-match section. In the post-ban period, an average of 3 (SD: 3.5) advertisements per-programme remained.

**Conclusions:**

A voluntary partial gambling advertising ban in the UK was associated with reductions in television advertising during live football games during the restricted period. There is evidence of increased advertising in the unrestricted period due to the partial nature of the ban. Future research is needed to explore the impact of the ban on other types of advertising, and across other channels.

## Introduction

Gambling is a public health issue (Korn et al. 2003; Public Health England (PHE), 2023; The Lancet 2017; Thomas et al. 2023). Harms span financial, emotional, health, economic, employment, and criminal harms: affecting individuals, families, and wider society (Langham et al. 2016; Wardle et al. 2018). A public health approach to gambling harm acknowledges a wider range of social and environmental risk factors (Korn et al. 2003), an important one being advertising (Public Health England (PHE), 2023).

Gambling advertising is omnipresent, concentrated around sports, and often represents complex and riskier bets (Deans et al. 2016; Newall et al. 2019; Torrance et al. 2021). It influences gambling behavior, with the greatest impact seen in more vulnerable populations, such as those who are higher risk gamblers (Bouguettaya et al. 2020; Killick et al. 2022; McGrane et al. 2023). Higher exposure to advertising is associated with increased urge to gamble, intentions to gamble, actual expenditure on gambling, and unplanned gambling spend (Russell et al. 2018; Browne et al. 2019; Roderique-Davies et al. 2020; Wardle et al. 2022). Qualitative literature suggests that it may act as a trigger to those in recovery (Binde 2009; Lopez-Gonzalez et al. 2020). Television (TV) advertising is often quoted as the most common type of exposure (IPSOS Mori 2020; Dunlop and Ballantyne 2021; Syvertsen et al. 2022). Whilst evidence linking exposure to advertising and harm is mostly indirect, Public Health England (PHE) identified advertising as a ‘societal’ risk factor for gambling harms (Public Health England (PHE), 2023).

The ‘gamblification’ of sport has received particular attention in recent years (Bunn et al. 2019; Sharman et al. 2020; Ireland et al. 2021; Hing et al. 2023). Football is the most popular sports to bet on in the UK, and is the most popular sport to watch globally (Ireland et al. 2019; The Gambling Commission 2023). Advertising around live football is ubiquitous: including TV advertisements, sponsorship, and pitch-side advertising (Cassidy and Ovenden 2017; Bunn et al. 2019; Ireland et al. 2021). There have been calls to restrict gambling advertising around live football (The Big Step 2021), given its potential to normalize gambling and influence gambling behavior and subsequent harms (Bouguettaya et al. 2020; Killick et al. 2022; McGrane et al. 2023).

European countries, such as Belgium (Belgian Official Gazette 2023) have committed to universal gambling advertising bans. Others have announced partial measures excluding online and ‘untargeted’ advertising (The Government of the Netherlands 2023). News outlets have imposed widespread bans on advertising in digital and print media (Waterson 2023). In the UK, gambling regulation comes under the 2005 Gambling Act (The UK Parliament 2005). Amongst other things, this act liberalized advertising laws, allowing the TV advertising of sports betting and casino products.

In the UK, gambling advertising is predominantly self-regulated by an industry body known as the Industry Group for Responsible Gambling, who enforce a voluntary code of conduct (Industry Group for Responsible Gambling 2023). Up to 2019, these voluntary codes prohibited TV advertising of the industry described ‘New Gambling Products’ (NGPs) – anything except lottery and bingo – during the watershed. In the UK, the watershed runs from 5:30am to 9:00pm. The only exemption to this were sports programmes. In August 2019, the industry group introduced a voluntary ‘Whistle-to-Whistle’ (W2W) ban during live sports programmes. Under this partial ban, gambling advertising was not permitted to appear within five minutes of the match beginning, until 5 minutes after the match had ended. This included during breaks-in-play where gambling advertising had been previously been prevalent (Ireland et al. 2021). The ban covered all live sports, excluding horse and dog racing. It was implemented for live sports during the watershed period only, and it did not cover other forms of advertising such as radio broadcasts, pitch-side hoardings, sponsorship of teams or leagues, or social media advertising. Other non-live sports programmes, such as sports documentaries or highlights programmes, were no longer exempt from the blanket watershed ban. Similar partial advertising bans have been implemented in Australia and Ireland (The Australian Communications and Media Authority 2021; The Irish Bookmakers Association 2021).

Despite many examples of advertising policies, there is a lack of comprehensive analysis of their impact. The UK Betting and Gaming Council (BGC), an industry body for gambling companies in the UK, reported a near elimination of TV gambling advertisements during the W2W period for all live sports programmes (The Betting and Gaming Council 2021). However, little is known about how the ban impacted advertising during programme sections outside of the W2W period, and advertising around specific types of sport. Analyzing changes in advertising at a granular level gives us a better understanding of how advertising bans affect the presence of advertisements on TV. Furthermore, in 2023 the UK government published its Gambling White Paper (Department for Culture and Media and Sport 2023) which, among other things, left advertising during sports to the discretion of sports governing bodies, and the industry. Therefore, it is imperative we understand how this self-regulation impacts the presence of advertising around live sports.

This study fills the evidence gap by exploring the change in the frequency and placement of gambling advertising following the introduction of the W2W ban in the UK. It focuses on live football given the high presence of advertising around this sport. It expands on the analysis by the UK gambling industry body to include more data, explore the impact by game section(Pre-game, 5-min before, Half-time, and Post-game), as well as over the total duration of live football games using matching models to reduce confounding.

## Materials and methods

### Data

The W2W ban was introduced on 1^st^ August 2019 (Industry Group for Responsible Gambling 2023). This study uses 3 months of data (1^st^ September to 1^st^ December) in the pre (2018) and post-ban (2019) years. To enhance comparability of the data, this study used the same time period at the beginning of the football season in the pre and post-ban period where the intensity of advertising was assumed to be similar. This also removed any potential variability in advertising due to irregular sporting events – those outside of the usual football calendar such as the World Cup.

Data were compiled from three sources: TV scheduling data (Concise Media), live kickoff times from a freely available online database (TVSportsGuide.com), and gambling advertising data (Nielsen Media). Information on the content of the three datasets is available in Appendix A, supplementary material. Kickoff data were scraped using ‘Selenium’ in R. A copy of the code used to scrape this data is available in Appendix B, supplementary material. Data were analyzed using STATA 17. The data covered all gambling advertising on all UK TV channels during the period studied

The datasets were restricted to live football programmes only, excluding live highlights programmes such as ‘Match of the Day’. The three datasets were combined and live games were collapsed into sections using approximate categories by minute of the live programme (Table 1). For each section of the live programme, the total number of gambling advertisements was calculated.

**Table 1.** Game sections.

Section	Description	Categorisation by minute
1	Pre-game	Up to 5 min before kickoff
2	5-min before	5 min before kickoff to 12 min after kickoff.
3	First-half	12 to 44 min after kickoff.
4	Half-time	45 to 74 min after kickoff.
5	Second-half	75 to 100 min after kickoff.
6	Post-game	Greater than 100 min after kickoff.

Due to varying game length, it was not possible to record exact end times of football games. Therefore, the post-game section was combined to include the post-game 5-min W2W period, as well as post-game programming. The wider window around Section 2 (5 min before) was to allow for late starting times. Sections 3 and 5 (First and Second Half) were included as a sense check; there should be no advertisements during the game play.

### Variables

The dependent variable was the frequency of advertisements during each section of the live football game, as well as the frequency over the total duration of the programme. The independent variable of interest was a binary variable representing the introduction of the W2W ban, equal to 1 if the year was 2019 (post-ban). Control variables included the day of the game, the channel (ITV, Sky, TNT Sports (formerly BT Sports), and other), and the time of the game. Channels categorized as ‘other’ included: S4C, Eurosport, and Viaplay Sports. Timings were categorized as midday (up to 12:59), early afternoon (13:00 to 16:59), early evening (17:00 to 18:59), and late evening (after 19:00).

### Statistical analysis

Regression models were run for game sections 1, 2, 4, 6, (Pre-game, 5-min before, Half-time, and Post-game) and the total duration of the live game. Linear models were first run, followed by Propensity Score (PSM) and Inverse Probability Weighted (IPW) matching models to reduce confounding. The latter models matched on the control variables stated above. PSM models matched treated (2019) and untreated (2018) football games based on a score generated using a regression of treatment against the aforementioned matching characteristics. This produced a score between 0 and 1 representing the probability of a game being ‘treated’. The model then matched football games which were close in propensity score, but differed in treatment. The ‘caliper’ indicates the total distance between the propensity scores of the matched football games; the wider the caliper, the less perfect the match. However, wider calipers can provide an appropriate approximation of a match, and can help to reduce confounding in the model. The minimum required caliper for these models was 0.4. Given that there is no agreed caliper suggested for use - research has suggested anywhere between 0.25 to 2 times the standard deviation of the logit of the propensity score (Stuart and Rubin 2008; Austin 2011) - IPW models were run for comparison. IPW matching is similar, but these models give a higher weight to treated football games (2019) that most resemble untreated football games (2018). This paper reports results from the IPW models which improve the balance of treatment and control groups to a greater extent than the linear and PSM models. Alternative model results are available in Appendix C, supplementary material.

## Ethics

Ethical approval was not required because this research used secondary advertising and TV scheduling data.

## Results

### Descriptive

Data covered 1049 live football games: 468 in 2018 and 581 in 2019 (Table 1). The average length of live programmes was 154 min in 2018, and 151 min in 2019. Games spanned across four broad categories of networks: ITV, Sky, TNT Sports, and Other. ITV is a commercial channel in the UK, whilst Sky and TNT Sports are subscription services. The majority of games occurred in the late evening, over the weekend, and on Sky or TNT Sports channels (Table 2).

**Table 2.** Descriptive statistics.

Variable		2018		2019		Total	
		*Freq*	*%*	*Freq*	*%*	*Freq*	*%*
Total number of matches		468	45%	581	55%	1049	100%
Total number of matches by channel							
	ITV	1	0.2%	7	1%	8	1%
	Sky	238	51%	249	43%	487	46%
	TNT	190	41%	198	34%	388	37%
	Other	39	8%	127	22%	166	16%
Total number of matches by day of the week							
	Monday	33	7%	46	8%	79	8%
	Tuesday	56	12%	56	10%	112	11%
	Wednesday	45	10%	53	9%	98	9%
	Thursday	46	10%	47	8%	93	9%
	Friday	60	13%	62	11%	122	12%
	Saturday	109	23%	128	22%	237	23%
	Sunday	119	25%	189	33%	308	29%
Total number of matches by time of day^a^							
	Midday	74	16%	114	20%	188	18%
	Early afternoon	70	15%	100	17%	170	16%
	Early evening	107	23%	126	22%	233	22%
	Late evening	217	46%	241	41%	458	44%
							
Total frequency of adverts		**2634**	**62%**	**1620**	**38%**	**4254**	**100%**
Total frequency of adverts by channel							
	ITV	18	1%	20	1%	38	1%
	Sky	2023	77%	1157	71%	3180	75%
	TNT Sports	580	22%	395	24%	975	23%
	Other	13	0%	48	3%	61	1%
							
		*Mean*	*SD*	*Mean*	*SD*	*Mean*	*SD*
Average adverts		5.8	5.30	2.9	3.50	4.2	4.60
							
Average programme length (mins)		154.90	32.70	151.60	33.10	152.9	32.70

^a^Midday (up to 12:59); Early afternoon (13:00 to 16:59); Early evening (17:00 to 18:59); Late evening (19:00 onwards).

**Figure 1.**
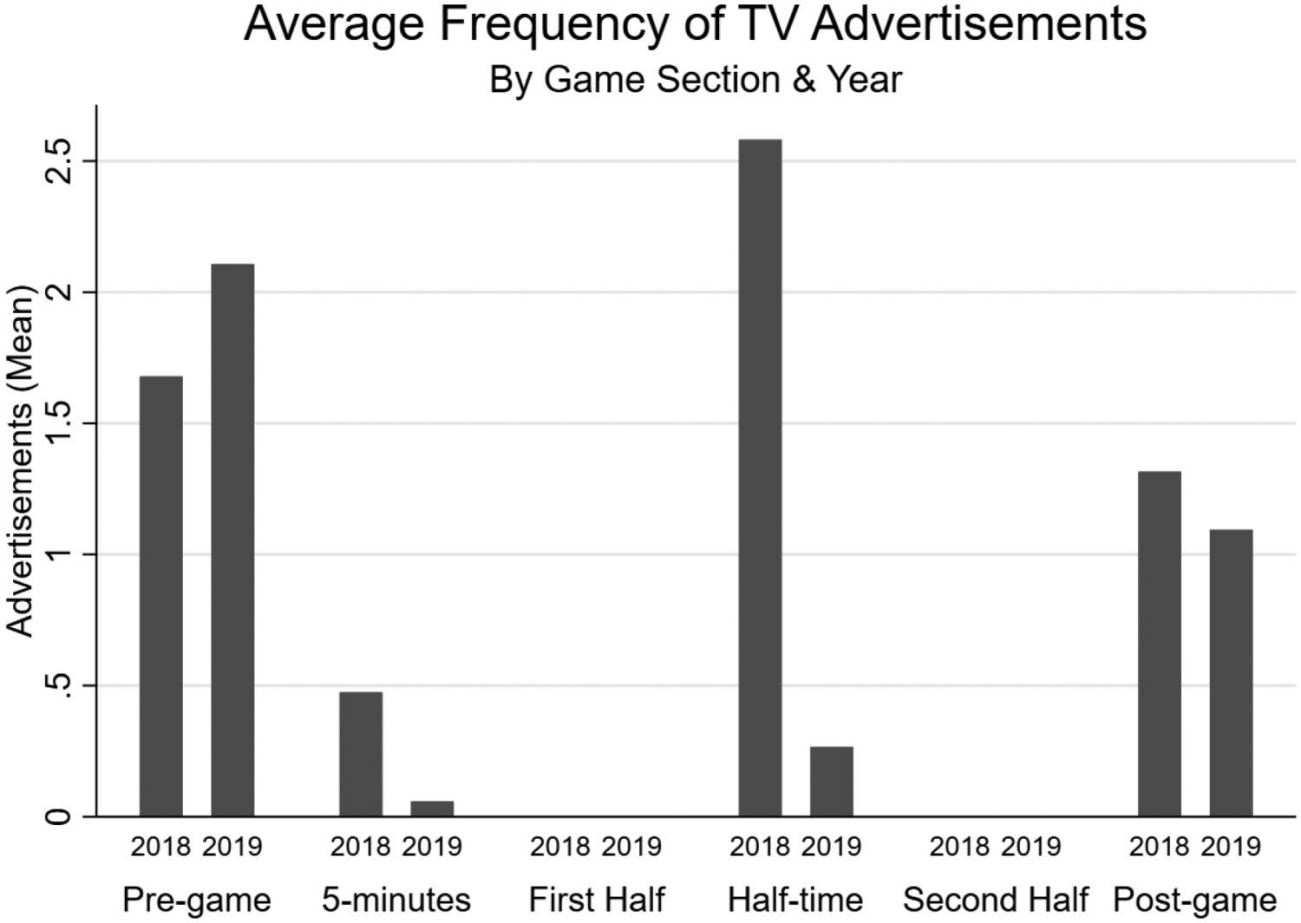
Average frequency of television gambling advertisements by game section and year.

There were an average of 5.8 advertisements per live football game in 2018, and 2.9 in 2019 (Table 2). A higher frequency of advertisements occurred on Sky channels. Figure 1 shows the number of advertisements by game section across the pre (2018) and post-ban (2019) years. There was a reduction in advertisements during the five minutes before the live game, and during half time section in 2019; the number of advertisements was still greater than 0 since lottery and bingo advertisements are permitted. There was an increase in advertisements in the pre-game section, and minimal change in the post-game section.

### IPW models

#### Results

Table 3 presents the IPW matching models. There was a reduction in advertisements equal to 2.3 advertisements (*p* < 0.001; CI[−2.75, −1.84]) per live game programme following the introduction of the W2W ban. The majority of this reduction occurred during half-time: 2.18 advertisements (*p* < 0.001; CI[−2.32, −2.04]), with a comparatively smaller reduction during the 5 min before the game (Table 3). There was an increase in advertising in the pre-game section of approximately 0.34 advertisements (*p* < 0.001; CI[0.09, 0.59]) per live game programme, and no change in the post-game section.

**Table 3.** Inverse probability weighted (IPW) matching model results.

	Pre-game	5-min before	Half-time	Post-game	Total Programme
**Post-ban (2019)**	**0.34** ***	**−0.39** ***	**−2.18** ***	**−0.01**	**−2.30** ***
	**[0.09,0.59]**	**[−0.44, −0.33]**	**[−2.32, −2.04]**	**[−0.22,0.20]**	**[−2.75, −1.84]**
Pre-ban (2018)	1.74***	0.45***	2.43***	1.21***	5.29***
	[1.55,1.93]	[0.40,0.50]	[2.30,2.57]	[1.05,1.37]	[4.88,5.70]
ITV (comparator)					
Sky	−1.93*	−2.03*	−2.03*	−2.03*	−2.02*
	[−3.98,0.12]	[−4.10,0.04]	[−4.11,0.05]	[−4.11,0.05]	[−4.09,0.05]
TNT Sports	−1.95*	−2.01*	−2.01*	−2.01*	−2.00*
	[−4.01,0.10]	[−4.08,0.07]	[−4.09,0.07]	[−4.09,0.07]	[−4.07,0.07]
Other	−2.27**	−0.87	−0.89	−0.89	−0.89
	[−4.39, −0.15]	[−2.97,1.22]	[−3.00,1.21]	[−3.00,1.21]	[−2.99,1.20]
Sunday (comparator)					
Monday	0.18	0.21	0.21	0.21	0.18
	[−0.53,0.88]	[−0.36,0.78]	[−0.36,0.78]	[−0.36,0.78]	[−0.39,0.75]
Tuesday	−0.18	−0.21	−0.21	−0.21	−0.23
	[−0.78,0.42]	[−0.71,0.30]	[−0.71,0.30]	[−0.71,0.30]	[−0.73,0.28]
Wednesday	−0.23	−0.06	−0.07	−0.07	−0.08
	[−0.87,0.41]	[−0.58,0.45]	[−0.58,0.45]	[−0.58,0.45]	[−0.60,0.44]
Thursday	−0.06	−0.28	−0.30	−0.30	−0.31
	[−0.70,0.58]	[−0.81,0.25]	[−0.83,0.24]	[−0.83,0.24]	[−0.84,0.23]
Friday	−0.30	−0.26	−0.23	−0.23	−0.28
	[−0.91,0.32]	[−0.76,0.25]	[−0.74,0.28]	[−0.74,0.28]	[−0.78,0.23]
Saturday	−0.07	−0.26	−0.30	−0.30	−0.28
	[−0.54,0.39]	[−0.64,0.12]	[−0.68,0.08]	[−0.68,0.08]	[−0.66,0.10]
Midday (comparator)^a^					
Early afternoon	−0.19	−0.14	−0.16	−0.16	−0.14
	[−0.78,0.40]	[−0.58,0.31]	[−0.61,0.29]	[−0.61,0.29]	[−0.59,0.31]
Early evening	−0.21	−0.22	−0.24	−0.24	−0.22
	[−0.74,0.31]	[−0.62,0.18]	[−0.64,0.17]	[−0.64,0.17]	[−0.62,0.18]
Late evening	−0.30	−0.37*	−0.40*	−0.40*	−0.35*
	[−0.86,0.25]	[−0.77,0.04]	[−0.81,0.02]	[−0.81,0.02]	[−0.76,0.06]
Constant	2.33**	2.42**	2.45**	2.45**	2.42**
	[0.24,4.42]	[0.33,4.51]	[0.35,4.54]	[0.35,4.54]	[0.33,4.51]
Observations	736	1049	1042	1042	1045

Models report unstandardized coefficients; 95% confidence intervals in brackets; ^a^Midday (up to 12:59); Early afternoon (13:00 to 16:59); Early evening (17:00 to 18:59); Late evening (19:00 onwards).

*p < 0.1, **p < 0.05, ***p < 0.01.

There were fewer advertisements on Sky and TNT Sports compared to ITV during the period studied, but these result did not reach standard levels of statistical significance (*p* > 0.05). For the results of the linear and PSM models, see Appendix C, supplementary material.

#### Model performance

**Figure 2.**
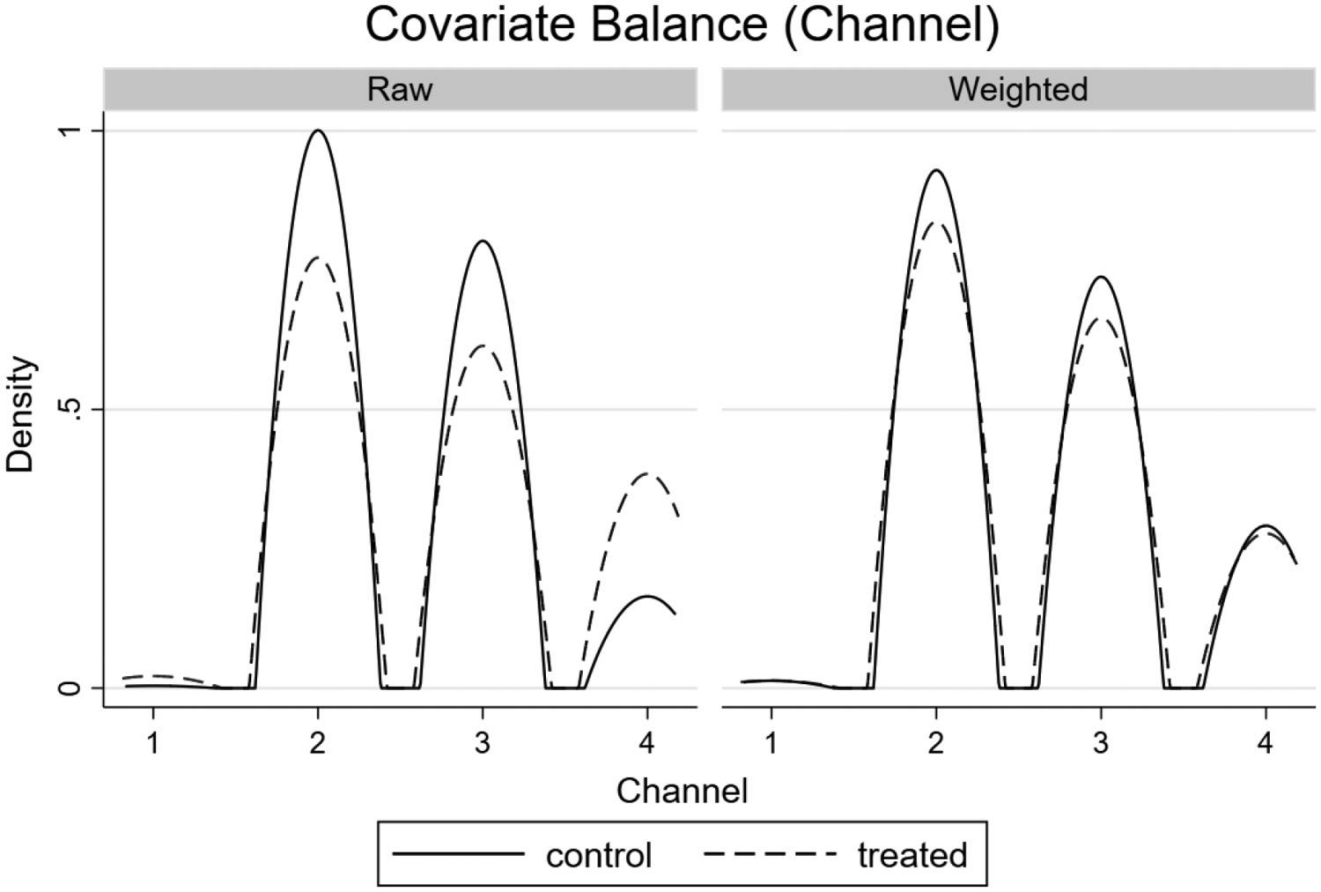
Density plot of covariate balance (channel the game was televised on) in the IPW models.

**Figure 3.**
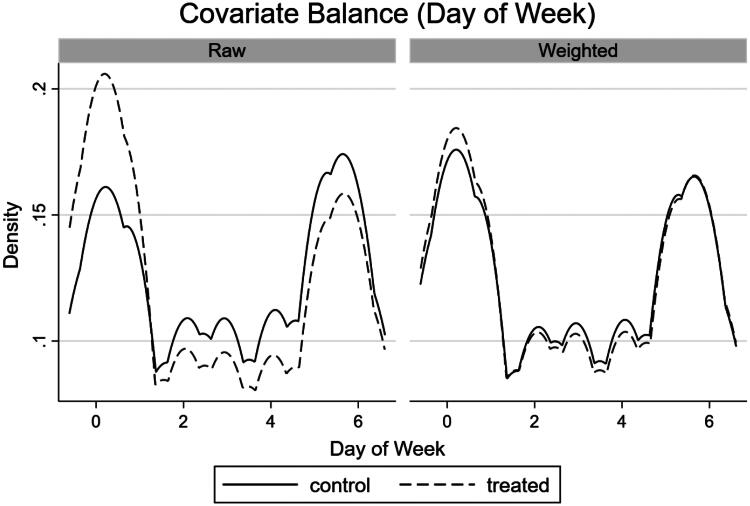
Density plot of covariate balance (day of the week the game was televised) in the IPW models.

**Figure 4.**
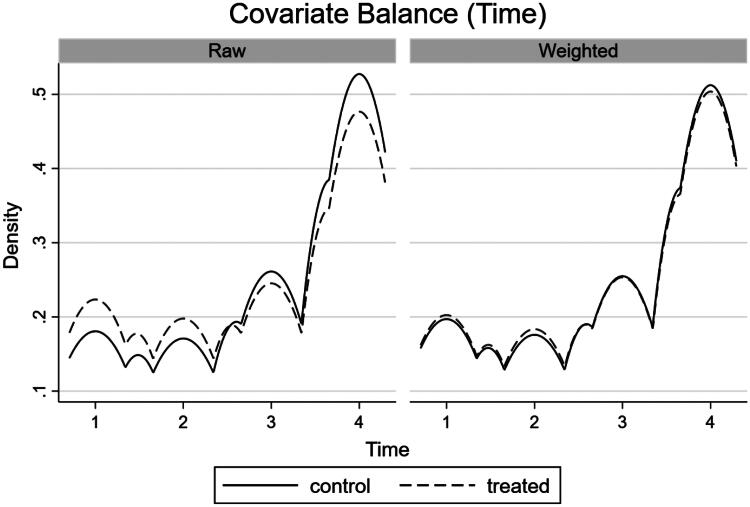
Density plot of covariate balance (time the game was televised) in the IPW models.

By observing the balance of covariates in the IPW model we can measure model performance. When covariates are balanced, their distribution does not differ between treatment (2019) and control (2018) groups, and therefore the groups are more comparable. Therefore, we want a matching model to provide balanced covariates. To explore this further, we have presented density plots (Figures 2–4) for the final IPW model (total programme). These are based on the differences in covariates between the treated and control groups for each of the three matching variables (channel, day of the week, time of the game). All three density plots show an improvement in fit in the weighted (matched) sample compared to the raw sample, indicating that the matched model has improved the balance of covariates. We can see this by the increased overlap of both density plots, bringing them close to complete overlap in the matched models. However, there is still significant overlap in the raw data models, likely due to the selection of two comparable periods at the beginning of the football season which improved the comparability of games in the pre and post-ban period.

We can formally test for covariate balance in the IPW model using a balance test, where the null hypothesis states that the matched model is balanced. Table 4 presents covariate balance statistics for each IPW model separately. The p-values of all models are greater than standard levels of statistical significance (*p* > 0.05), so we fail to reject the null hypothesis; the matched models are all balanced. Table 4 also indicates that the number of observations between the treated and control groups have become more balanced in the matched sample, compared to the raw sample. This removes any bias that may occur due to the increased number of games in the treated group (2019) versus the control group (2018).

**Table 4.** Balance of covariates in IPW models.

	Observations^a^				Balance Test^b^
	Raw		Weighted		
	Treated	Control	Treated	Control	*p*-value
Pre-game	381	355	368.2	367.8	0.98
5-min before	581	468	527	522	0.21
Half-time	576	466	523.4	518.6	0.21
Post-game	576	466	523.4	518.6	0.21
Total programme	578	467	524.9	520.1	0.22

^a^The number of observations is the number of live games; ^b^Balance test is a Chi-Squared test where H0:Covariates are balanced between treatment and control groups in the IPW model.

Appendix D, supplementary material reports detailed covariate balance tables for both the PSM and IPW models. For covariates to be well-balanced, the matched standardized mean difference should be close to zero, and the matched variance ratios should be close to 1. These tables show that the IPW models balance the covariates marginally better than the PSM models. For further information, see Appendix D, supplementary material.

## Discussion

This study examined the change in the frequency and placement of gambling advertising during live televised football as a result of an industry-implemented partial advertising ban around live sports broadcasts in the UK. It found that the ban led to an overall reduction in TV advertisements around live football games during the restricted periods (5-min before, and Half-time), with a small increase in the unrestricted pre-match section and no change in the post-match section. The comparatively smaller decreases seen during the 5 min before the game compared to half-time were likely due to the smaller time frame available for advertisements. In 2019, there remained an average of 3 gambling advertisements per live football game, attributable to the partial nature of the ban which does not apply to the pre or post-match period. Previous research has reported an average of 4.5 advertisements per game during the Men’s 2020 Euro tournament, and 5.2 gambling advertisements per game during 2022 Qatar World Cup (Newall et al. 2022; Sharman et al. 2023). This is likely due to their study of large sporting events, which this study does not cover. Other research highlights a noticeable presence of gambling advertising through other forms (including pitch-side and sponsorship) during live televised football (Cassidy and Ovenden 2017; Purves et al. 2020; Rossi et al. 2023; Torrance et al. 2023), likely resulting from the exclusion of these other forms of advertising from this ban. Therefore, TV advertising restrictions may be an effective policy tool for reducing the frequency of gambling advertisements on TV around live football games. However, partial bans may be less effective in reducing the overall prevalence of advertisements on TV.

### Strengths

This paper used three rich datasets on TV schedules, kickoff times, and gambling advertising to examine the impact of a voluntary advertising ban on the presence of advertising during live televised football. It goes beyond the analysis by the UK gambling industry body by looking at football specifically, over a longer period of time, and including additional data. We used matching models to reduce confounding and identify the independent effect of the ban.

### Limitations

We did not have data on advertising through other channels such as direct, online, pitch-side, or sponsorship. There might be unintended consequences if the industry increased other forms of advertising to compensate for losses in TV advertising. Evidence shows that advertisements are still highly prevalent in these areas (Purves et al. 2020; Torrance et al. 2023). Advertising may also have changed across the rest of the TV network; this is an area requiring further research.

### Policy implications

A voluntary partial gambling advertising ban in the UK was associated with a reduction in gambling advertising around live football games in 2019. Reductions in advertising during the 5 min before the game, and at half-time, are similar to those reported by the industry body in the UK. However, reductions over the total duration of live football may be lower than the 78% reduction reported for all live sport (The Betting and Gaming Council 2021): potentially only around 43%. An important finding is that the industry did not substitute advertisements during the restricted period for lottery and bingo advertisements, which are permitted. Results indicate that there may have been some spreading of television advertisements into the pre-match (unrestricted) section, although the magnitude of this effect is comparatively smaller.

There is no evidence to suggest that the five minutes before and after a live game is the optimal window to restrict gambling advertising. A cognitive theory known as the Serial Positioning Effect (Glanzer and Cunitz 1966) explains how people are more likely to recall items seen at the beginning and the end of list, rather than the middle. Applied to this context, it may suggest that people may be more likely to recall advertisements in the pre and post-game section, which are areas of unrestricted advertising. Whilst we are unable to comment on this in the current study, this is an area that would benefit from further research

Evidence from other industries, such as alcohol and tobacco, indicate that partial advertising bans are less effective than universal bans (Braverman and Aarø 2004; Kovic et al. 2018; Potvin Kent and Pauzé 2018; Boyland et al. 2022). There may be increases in other types of advertising, which reduce their impact. Online platforms provide an opportunity since these are highly unregulated, and have wide reach (Hastings et al. 2010; Rossi and Nairn 2022). Gamble Aware reported that the gambling industry spent 15% of their advertising budget on TV, and 10% on online advertising in 2017. Spend on social media had increased by 52% per annum between 2014 and 2017. Online marketing was reported to have increased by 23% per annum over the same period (Gamble Aware 2018). Ipsos MORI estimated TV spend to be £193,548,007, and online impressions at £8,942,818 (IPSOS Mori 2020). It is likely that online gambling advertising has modified in line with technological changes in the UK over the last seven years, although we do not have the data required to explore this.

Future research should comprehensively explore how the introduction of specific restrictions impacts overall exposure to advertising across various advertising channels. For example, it should look at how the W2W ban impacted other live sports, including those not subject to the ban (horse racing). It should also examine the entire TV network to assess whether there were changes in advertising around other TV genres, such as entertainment. The impact of the window of restriction needs further research, in addition to looking at how overall changes in advertising translate to a change in gambling behavior.

## Conclusions

This study illustrates that partial restrictions on gambling advertising during televised live sports were associated with a reduction in the number of advertisements across live football games during the restricted period, and some increases during the unrestricted period. Following the ban, advertisements remained prevalent during live football. This may impact the ability of the ban to reduce harm, since partial bans are known to be less effective. Future research must look at the wider impact of the ban, including whether there is any change in advertising during other programming post-watershed, or other forms of advertising. Evidence on the subsequent behavioral impact of the ban is also required.

## Supplementary Material

Supplemental Material

## Data Availability

Advertising data used in this study is not available for sharing due to licensing restrictions. However, STATA do-files and log-files are available on request from the corresponding author (EM), in addition to the scraped kickoff dataset.
