# Supplementary material for: How did the ‘whistle-to-whistle’ ban affect gambling advertising on TV? A live football matching study
Source: Addict Res Theory. 2024 May 20;33(2):134–42. doi: 10.1080/16066359.2024.2355183 (PMC11934951; doi:10.1080/16066359.2024.2355183)
Supplement: Supplemental Material [file IART_A_2355183_SM8075.docx]

**APPENDIX A: DATA SPECIFICATION**

*APPENDIX A Table 1: Data Specification*

| **Specification** | **Concise Media Co (1)** | **TVSportsGuide (2)** | **Nielsen Media Ltd (3)** |
| --- | --- | --- | --- |
| *Date* | ✓ | ✓ | ✓ |
| *Start time of the programme* | ✓ |  |  |
| *End time of the programme* | ✓ |  |  |
| *Kick-off time for the live game* |  | ✓ |  |
| *Time of the advert* |  |  | ✓ |
| *Holding company* |  |  | ✓ |
| *Advertiser* |  |  | ✓ |
| *Channel* | ✓ | ✓ | ✓ |
| *Programme name* | ✓ | ✓ | ✓ |
| *Programme genre* | ✓ |  | ✓ |
| *Subgenre (including type of sport)* |  | ✓ | ✓ |
| *Sports League* |  | ✓ |  |

**APPENDIX B: ‘RSELENIUM’ CODE FOR SCRAPING KICKOFF DATA**

###############################################################################

############################LOAD PACKAGES###################################

**#load relevant packages**

library(RSelenium)

library(tidyverse)

library(rvest)

library(writexl)

library(dplyr)

###############################################################################

#######################SELENIUM DRIVER SETUP###############################

**#set up selenium driver**

rD <- rsDriver(browser="firefox", port=12246L, verbose=F)

remDr <- rD[["client"]]

baseurl <- ("https://www.tvsportguide.com/archive/")

###############################################################################

#############################FUNCTIONS#######################################

**# Create a list of dates for the periods required - YYYY-MM-DD**

**#1st Sept to 1st Dec 2018 and 2019**

date_seq <- c(seq(from = as.Date("2018-09-01"), to = as.Date("2018-12-01"), by = "days"),

seq(from = as.Date("2019-09-01"), to = as.Date("2019-12-01"), by = "days"))

**#empty dataframe for elements**

elements_df <- data.frame(Date = character(0),

Time = character(0),

Title = character(0),

Sport = character(0),

League = character(0),

Channel1 = character(0),

Channel2 = character(0),

Channel3 = character(0),

Channel4 = character(0))

**#function to scrape data (by element)**

data_scrape <- function (date){

**#some matches have "hidden times" and show the score instead of time on screen. Need time data (find hidden time text).**

time <- matchlist[[n]]$findChildElement(using = "tag name", value = "time")

Time <-as.character(time$getElementText())

is_time <- grepl(":", Time)

if (is_time == FALSE){

hiddentime<- matchlist[[n]]$findChildElement(using = "class", value = "hidden-time")

Time <- as.character(hiddentime$getElementAttribute("textContent"))

}

if (is_time == TRUE) {

Time <- as.character(time$getElementText())

}

**#match title**

title <- matchlist[[n]]$findChildElement(using = "tag name", value = "h3")

Title <- as.character(title$getElementText())

**#sport**

sport <- matchlist[[n]]$findChildElement(using = "class", value = "sicon")

Sport<- as.character(sport$getElementAttribute("Title"))

**#league (some do not have a league so skip if empty)**

league <- matchlist[[n]]$findChildElements(using = "class", value = "league")

if (length(league) != 0) {

league <- matchlist[[n]]$findChildElement(using = "class", value = "league")

League <- as.character(league$getElementText())

}

if (length(league) == 0) {

League <- ""

}

#channels

Channel1 <- NA

Channel2 <- NA

Channel3 <- NA

Channel4 <- NA

channels <- channellist[[n]]$findChildElements(using = "tag name", value = "a")

for(n in 1:length(channels)){

if (length(channels) == 0) {

Channel1 <- NA

}

if (length(channels) != 0) {

assign(paste("Channel", n, sep=""), as.character(channels[[n]]$getElementAttribute("title")))

}

}

**#current date (in the loop)**

Date <- as.character(format(date_seq[i], format="%Y/%m/%d"))

**#dataframe**

df <- data.frame(Date = Date,

Time = Time,

Title = Title,

Sport = Sport,

League = League,

Channel1 = Channel1,

Channel2 = Channel2,

Channel3 = Channel3,

Channel4 = Channel4)

return(df)

}

###############################################################################

#############################DATA SCRAPE LOOP###############################

**#loop round dates and matches**

for (i in 1:(length(date_seq))) {

url <- paste0(baseurl, (gsub("/0", "/", format(date_seq[i], format="/%Y/%m/%d"))))

remDr$navigate(url)

matchlist<- remDr$findElements(using="class", value="match")

for (n in 1:length(matchlist)) {

channellist <- remDr$findElements(using="class", value="channels")

elements_df <- rbind(elements_df, data_scrape(date_seq[i]))

}

}

**#close selenium server**

remDr$close()

rD$server$stop()

###############################################################################

########################EDIT AND SAVE DATASET###############################

**#reorder columns**

elements_df[,c("Date", "Time", "Title", "Sport", "League", "Channel1", "Channel2", "Channel3", "Channel4")]

**#fill in missing values with 0 (only for league - channels already assigned NA)**

elements_df[elements_df == ""] <- 0

**#duplicate rows 4 times**

full_data_mod <- elements_df %>%

expand_grid(Channel = c("Channel1", "Channel2", "Channel3", "Channel4"))

**#replace NA with missing (to allow the following loop to run)**

full_data_mod[is.na(full_data_mod)] <- ""

**#fill in the value of channel for each row**

for( i in 1:nrow(full_data_mod)){

if(full_data_mod$Channel[i]=="Channel1") {

full_data_mod$Channel[i] <- full_data_mod$Channel1[i]

}

if(full_data_mod$Channel[i]=="Channel2") {

full_data_mod$Channel[i] <- full_data_mod$Channel2[i]

}

if(full_data_mod$Channel[i]=="Channel3") {

full_data_mod$Channel[i] <- full_data_mod$Channel3[i]

}

if(full_data_mod$Channel[i]=="Channel4") {

full_data_mod$Channel[i] <- full_data_mod$Channel4[i]

}

}

**#drop unwanted channel columns**

clean_data <- subset(full_data_mod, select = -6:-9)

**#assign missing values <- NA again**

clean_data[clean_data == ""] <- NA

**#drop unwanted rows (each match has 4 rows but not all matches have 4 channels)**

**#now have a row for each match (duplicated for each additional channel it is shown on)**

final_data <- drop_na(clean_data)

###############################################################################

############################SAVE FULL DATASET################################

**#save new dataset**

**#set working directory and save dataset to relevant folder**

setwd("C:/Users/ellen/OneDrive/PhD/Gambling/Secondary Data Analysis/Web Scraping in R")

save(final_data ,file="CompleteLoopData_EM.Rda")

**#export dataset to excel**

write_xlsx(final_data,"C:/Users/ellen/OneDrive/PhD/Gambling/Secondary Data Analysis/Web Scraping in R\\Complete_Loop_Data_EM_XL.xlsx")

**APPENDIX C: ALTERNATIVE REGRESSION MODEL RESULTS**

**Appendix C Table 1: Linear Regression Results**

|  | Pre-game | 5-minutes before | Half-time | Post-game | Total Programme |
| --- | --- | --- | --- | --- | --- |
| **Post-ban (2019)** | **0.35^***^** | **-0.39^***^** | **-2.24^***^** | **0.01** | **-2.31^***^** |
|  | **[0.10,0.60]** | **[-0.45,-0.34]** | **[-2.38,-2.10]** | **[-0.20,0.22]** | **[-2.77,-1.86]** |
|  |  |  |  |  |  |
| ITV (comparator) |  |  |  |  |  |
|  |  |  |  |  |  |
| Sky | 0.38 | -0.17 | 0.41 | 0.63 | 1.09 |
|  | [-0.83,1.59] | [-0.48,0.14] | [-0.37,1.20] | [-0.56,1.82] | [-1.48,3.66] |
|  |  |  |  |  |  |
| TNT Sports | -0.68 | -0.35^**^ | -0.58 | -0.79 | -2.78^**^ |
|  | [-1.90,0.54] | [-0.66,-0.04] | [-1.37,0.21] | [-1.98,0.41] | [-5.36,-0.20] |
|  |  |  |  |  |  |
| Other | -1.77^***^ | -0.44^***^ | -0.65 | -1.34^**^ | -4.35^***^ |
|  | [-3.07,-0.47] | [-0.76,-0.12] | [-1.45,0.14] | [-2.55,-0.14] | [-6.96,-1.74] |
|  |  |  |  |  |  |
| Sunday (comparator) |  |  |  |  |  |
|  |  |  |  |  |  |
| Monday | 1.72^***^ | -0.07 | 0.17 | 0.90^***^ | 1.83^***^ |
|  | [1.11,2.32] | [-0.20,0.05] | [-0.14,0.49] | [0.42,1.37] | [0.80,2.86] |
|  |  |  |  |  |  |
| Tuesday | 0.72^***^ | -0.07 | -0.13 | 0.19 | 0.34 |
|  | [0.21,1.23] | [-0.18,0.03] | [-0.40,0.14] | [-0.22,0.60] | [-0.54,1.22] |
|  |  |  |  |  |  |
| Wednesday | 1.16^***^ | 0.04 | -0.12 | 0.52^**^ | 1.00^**^ |
|  | [0.61,1.71] | [-0.07,0.15] | [-0.40,0.16] | [0.10,0.94] | [0.09,1.91] |
|  |  |  |  |  |  |
| Thursday | 0.50^*^ | -0.20^***^ | -0.11 | 0.47^**^ | 0.23 |
|  | [-0.05,1.04] | [-0.31,-0.09] | [-0.39,0.17] | [0.05,0.90] | [-0.68,1.15] |
|  |  |  |  |  |  |
| Friday | 0.78^***^ | 0.10^*^ | -0.17 | 0.15 | 0.43 |
|  | [0.26,1.31] | [-0.01,0.20] | [-0.45,0.10] | [-0.26,0.57] | [-0.46,1.31] |
|  |  |  |  |  |  |
| Saturday | 0.13 | 0.12^***^ | -0.12 | 0.50^***^ | 0.66^*^ |
|  | [-0.27,0.53] | [0.04,0.20] | [-0.33,0.08] | [0.18,0.81] | [-0.01,1.34] |
|  |  |  |  |  |  |
| Midday (comparator) |  |  |  |  |  |
|  |  |  |  |  |  |
| Early afternoon | -0.28 | 0.04 | -0.17 | 0.18 | 0.13 |
|  | [-0.78,0.23] | [-0.06,0.13] | [-0.42,0.07] | [-0.19,0.55] | [-0.68,0.93] |
|  |  |  |  |  |  |
| Early evening | -1.06^***^ | -0.03 | -0.14 | 0.07 | -0.34 |
|  | [-1.50,-0.62] | [-0.12,0.05] | [-0.36,0.07] | [-0.26,0.41] | [-1.06,0.38] |
|  |  |  |  |  |  |
| Late evening | -1.06^***^ | 0.00 | 0.06 | 0.81^***^ | 0.82^**^ |
|  | [-1.53,-0.58] | [-0.08,0.09] | [-0.16,0.28] | [0.48,1.15] | [0.10,1.53] |
|  |  |  |  |  |  |
| Constant | 2.17^***^ | 0.73^***^ | 2.77^***^ | 0.69 | 5.76^***^ |
|  | [0.88,3.46] | [0.41,1.05] | [1.96,3.58] | [-0.54,1.93] | [3.09,8.42] |
| Observations | 736 | 1049 | 1042 | 1042 | 1045 |

*Models report unstandardised coefficients; 95% confidence intervals in bracket; Midday (up to 12:59); Early afternoon (13:00 to 16:59); Early evening (17:00 to 18:59); Late evening (19:00 onwards).*

*^*^ p < 0.1, ^**^ p < 0.05, ^***^ p < 0.01*

**Appendix C Table 2: Propensity Score Matching (PSM) Results**

|  | Pre-game | 5-minutes before | Half-time | Post-game | Total Programme |
| --- | --- | --- | --- | --- | --- |
| **Post-ban (2019)** | **0.34^***^** | **-0.38^***^** | **-2.16^***^** | **0.01** | **-2.20^***^** |
|  | **[0.10,0.58]** | **[-0.45,-0.32]** | **[-2.32,-1.99]** | **[-0.20,0.21]** | **[-2.64,-1.76]** |
| Observations | 736 | 1049 | 1042 | 1042 | 1045 |

*Models report unstandardised coefficients; 95% confidence intervals in brackets*

*^*^ p < 0.1, ^**^ p < 0.05, ^***^ p < 0.01*

**APPENDIX D: COVARIATE BALANCE TABLES FOR MATCHING MODELS**

**Appendix D Table 1: Covariate Balance Tables for Inverse Probability Weighted (IPW) Matching Models**

|  | **Pre-game** | | | |  | **5-minutes before** | | | |  | **Half-time** | | | |  | **Post-game** | | | |  | **Total programme** | | | |
| --- | --- | --- | --- | --- | --- | --- | --- | --- | --- | --- | --- | --- | --- | --- | --- | --- | --- | --- | --- | --- | --- | --- | --- | --- |
| **Matching Variables** | ***St Diff*** | | ***Var Ratio*** | |  | ***St Diff*** | | ***Var Ratio*** | |  | ***St Diff*** | | ***Var Ratio*** | |  | ***St Diff*** | | ***Var Ratio*** | |  | ***St Diff*** | | ***Var Ratio*** | |
|  | *Raw* | *Match* | *Raw* | *Match* |  | *Raw* | *Match* | *Raw* | *Match* |  | *Raw* | *Match* | *Raw* | *Match* |  | *Raw* | *Match* | *Raw* | *Match* |  | *Raw* | *Match* | *Raw* | *Match* |
| ***Channel*** |  |  |  |  |  |  |  |  |  |  |  |  |  |  |  |  |  |  |  |  |  |  |  |  |
| Sky | 0.04 | 0.00 | 0.99 | 1.00 |  | -0.16 | -0.01 | 0.98 | 1.00 |  | -0.16 | -0.01 | 0.98 | 1.00 |  | -0.16 | -0.01 | 0.98 | 1.00 |  | -0.15 | -0.01 | 0.98 | 1.00 |
| TNT Sports | -0.02 | 0.00 | 0.99 | 1.00 |  | -0.13 | -0.01 | 0.93 | 1.00 |  | -0.13 | -0.01 | 0.93 | 1.00 |  | -0.13 | -0.01 | 0.93 | 1.00 |  | -0.13 | -0.01 | 0.93 | 1.00 |
| Other | -0.09 | 0.00 | 0.72 | 0.99 |  | 0.38 | 0.02 | 2.24 | 1.04 |  | 0.38 | 0.02 | 2.20 | 1.04 |  | 0.38 | 0.02 | 2.20 | 1.04 |  | 0.37 | 0.02 | 2.20 | 1.04 |
| ***Day of the week*** |  |  |  |  |  |  |  |  |  |  |  |  |  |  |  |  |  |  |  |  |  |  |  |  |
| Monday | 0.06 | 0.00 | 1.22 | 1.01 |  | 0.03 | 0.01 | 1.11 | 1.03 |  | 0.03 | 0.01 | 1.12 | 1.03 |  | 0.03 | 0.01 | 1.12 | 1.03 |  | 0.03 | 0.01 | 1.11 | 1.03 |
| Tuesday | -0.05 | 0.00 | 0.88 | 1.00 |  | -0.07 | -0.01 | 0.83 | 0.98 |  | -0.07 | -0.01 | 0.83 | 0.98 |  | -0.07 | -0.01 | 0.83 | 0.98 |  | -0.07 | -0.01 | 0.83 | 0.98 |
| Wednesday | -0.07 | 0.00 | 0.82 | 0.99 |  | -0.02 | -0.01 | 0.95 | 0.96 |  | -0.02 | -0.01 | 0.96 | 0.96 |  | -0.02 | -0.01 | 0.96 | 0.96 |  | -0.02 | -0.01 | 0.96 | 0.96 |
| Thursday | 0.00 | 0.00 | 1.00 | 1.01 |  | -0.06 | -0.02 | 0.84 | 0.95 |  | -0.07 | -0.02 | 0.83 | 0.95 |  | -0.07 | -0.02 | 0.83 | 0.95 |  | -0.07 | -0.02 | 0.82 | 0.95 |
| Friday | -0.09 | -0.01 | 0.80 | 0.97 |  | -0.07 | -0.01 | 0.85 | 0.97 |  | -0.06 | -0.01 | 0.87 | 0.97 |  | -0.06 | -0.01 | 0.87 | 0.97 |  | -0.07 | -0.01 | 0.85 | 0.98 |
| Saturday | 0.03 | 0.00 | 1.03 | 1.00 |  | -0.03 | 0.00 | 0.96 | 1.01 |  | -0.04 | 0.00 | 0.94 | 1.00 |  | -0.04 | 0.00 | 0.94 | 1.00 |  | -0.04 | 0.00 | 0.95 | 1.00 |
| ***Time of game*** |  |  |  |  |  |  |  |  |  |  |  |  |  |  |  |  |  |  |  |  |  |  |  |  |
| Early afternoon | 0.04 | 0.00 | 1.08 | 1.00 |  | 0.06 | 0.02 | 1.12 | 1.03 |  | 0.06 | 0.02 | 1.12 | 1.03 |  | 0.06 | 0.02 | 1.12 | 1.03 |  | 0.06 | 0.02 | 1.12 | 1.03 |
| Early evening | 0.00 | 0.00 | 1.00 | 1.00 |  | -0.03 | 0.00 | 0.96 | 0.99 |  | -0.03 | -0.01 | 0.95 | 0.99 |  | -0.03 | -0.01 | 0.95 | 0.99 |  | -0.04 | 0.00 | 0.95 | 0.99 |
| Late evening | -0.10 | 0.00 | 0.99 | 1.00 |  | -0.10 | -0.02 | 0.98 | 1.00 |  | -0.10 | -0.02 | 0.98 | 1.00 |  | -0.10 | -0.02 | 0.98 | 1.00 |  | -0.09 | -0.02 | 0.98 | 1.00 |

**For covariates to be well-balanced, standardised mean differences (St Diff) should be close to 0 in the matched column; variance ratios (Var Ratio) should be close to 1 in the matched column*

**Appendix D Table 2: Covariate Balance Tables for Propensity Score Matching (PSM) Models**

|  | **Pre-game** | | | |  | **5-minutes before** | | | |  | **Half-time** | | | |  | **Post-game** | | | |  | **Total programme** | | | |
| --- | --- | --- | --- | --- | --- | --- | --- | --- | --- | --- | --- | --- | --- | --- | --- | --- | --- | --- | --- | --- | --- | --- | --- | --- |
| **Matching Variables** | ***St Diff*** | | ***Var Ratio*** | |  | ***St Diff*** | | ***Var Ratio*** | |  | ***St Diff*** | | ***Var Ratio*** | |  | ***St Diff*** | | ***Var Ratio*** | |  | ***St Diff*** | | ***Var Ratio*** | |
|  | *Raw* | *Match* | *Raw* | *Match* |  | *Raw* | *Match* | *Raw* | *Match* |  | *Raw* | *Match* | *Raw* | *Match* |  | *Raw* | *Match* | *Raw* | *Match* |  | *Raw* | *Match* | *Raw* | *Match* |
| ***Channel*** |  |  |  |  |  |  |  |  |  |  |  |  |  |  |  |  |  |  |  |  |  |  |  |  |
| Sky | 0.04 | -0.03 | 0.99 | 1.01 |  | -0.16 | 0.00 | 0.98 | 1.00 |  | -0.16 | 0.00 | 0.98 | 1.00 |  | -0.16 | 0.00 | 0.98 | 1.00 |  | -0.15 | 0.00 | 0.98 | 1.00 |
| TNT Sports | -0.02 | 0.01 | 0.99 | 1.01 |  | -0.13 | 0.00 | 0.93 | 1.00 |  | -0.13 | 0.00 | 0.93 | 1.00 |  | -0.13 | 0.00 | 0.93 | 1.00 |  | -0.13 | 0.00 | 0.93 | 1.00 |
| Other | -0.09 | 0.04 | 0.72 | **1.16** |  | 0.38 | 0.00 | 2.24 | 1.00 |  | 0.38 | 0.00 | 2.20 | 1.00 |  | 0.38 | 0.00 | 2.20 | 1.00 |  | 0.37 | 0.00 | 2.20 | 1.00 |
|  |  |  |  |  |  |  |  |  |  |  |  |  |  |  |  |  |  |  |  |  |  |  |  |  |
| ***Day of the week*** |  |  |  |  |  |  |  |  |  |  |  |  |  |  |  |  |  |  |  |  |  |  |  |  |
| Monday | 0.06 | 0.02 | 1.22 | 1.07 |  | 0.03 | 0.03 | 1.11 | 1.09 |  | 0.03 | 0.03 | 1.12 | 1.09 |  | 0.03 | 0.03 | 1.12 | 1.09 |  | 0.03 | 0.03 | 1.11 | 1.09 |
| Tuesday | -0.05 | 0.03 | 0.88 | 1.06 |  | -0.07 | 0.00 | 0.83 | 1.00 |  | -0.07 | 0.00 | 0.83 | 1.00 |  | -0.07 | 0.00 | 0.83 | 1.00 |  | -0.07 | 0.00 | 0.83 | 1.00 |
| Wednesday | -0.07 | -0.02 | 0.82 | 0.93 |  | -0.02 | -0.04 | 0.95 | 0.91 |  | -0.02 | -0.03 | 0.96 | 0.92 |  | -0.02 | -0.03 | 0.96 | 0.92 |  | -0.02 | -0.04 | 0.96 | 0.90 |
| Thursday | 0.00 | 0.07 | 1.00 | **1.25** |  | -0.06 | 0.01 | 0.84 | 1.04 |  | -0.07 | -0.01 | 0.83 | 0.96 |  | -0.07 | -0.01 | 0.83 | 0.96 |  | -0.07 | 0.01 | 0.82 | 1.04 |
| Friday | -0.09 | -0.07 | 0.80 | **0.86** |  | -0.07 | -0.04 | 0.85 | 0.90 |  | -0.06 | -0.02 | 0.87 | 0.95 |  | -0.06 | -0.02 | 0.87 | 0.95 |  | -0.07 | -0.04 | 0.85 | 0.90 |
| Saturday | 0.03 | -0.01 | 1.03 | 0.99 |  | -0.03 | 0.04 | 0.96 | 1.06 |  | -0.04 | 0.04 | 0.94 | 1.05 |  | -0.04 | 0.04 | 0.94 | 1.05 |  | -0.04 | 0.04 | 0.95 | 1.06 |
|  |  |  |  |  |  |  |  |  |  |  |  |  |  |  |  |  |  |  |  |  |  |  |  |  |
| ***Time of game*** |  |  |  |  |  |  |  |  |  |  |  |  |  |  |  |  |  |  |  |  |  |  |  |  |
| Early aftern~n | 0.04 | 0.02 | 1.08 | 1.04 |  | 0.06 | 0.04 | 1.12 | 1.07 |  | 0.06 | 0.06 | 1.12 | **1.12** |  | 0.06 | 0.06 | 1.12 | **1.12** |  | 0.06 | 0.04 | 1.12 | 1.08 |
| Early evening | 0.00 | 0.00 | 1.00 | 1.00 |  | -0.03 | -0.07 | 0.96 | 0.92 |  | -0.03 | -0.05 | 0.95 | 0.94 |  | -0.03 | -0.05 | 0.95 | 0.94 |  | -0.04 | -0.07 | 0.95 | 0.92 |
| Late evening | -0.10 | -0.01 | 0.99 | 1.00 |  | -0.10 | 0.01 | 0.98 | 1.00 |  | -0.10 | -0.01 | 0.98 | 1.00 |  | -0.10 | -0.01 | 0.98 | 1.00 |  | -0.09 | 0.01 | 0.98 | 1.00 |

**For covariates to be well-balanced, standardised mean differences (St Diff) should be close to 0 in the matched column; variance ratios (Var Ratio) should be close to 1 in the matched column*
